# Wearable Sensors for Respiration Monitoring: A Review

**DOI:** 10.3390/s23177518

**Published:** 2023-08-30

**Authors:** Tauseef Hussain, Sana Ullah, Raúl Fernández-García, Ignacio Gil

**Affiliations:** 1 Department of Electronic Engineering, Universitat Politècnica de Catalunya, 08222 Terrassa, Spain; raul.fernandez-garcia@upc.edu (R.F.-G.); ignasi.gil@upc.edu (I.G.); 2 Department of Electrical and Information Engineering, Politecnico di Bari, 70126 Bari, Italy; s.ullah1@phd.poliba.it

**Keywords:** respiration sensors, breathing sensors, flexible sensors, wearable sensors

## Abstract

This paper provides an overview of flexible and wearable respiration sensors with emphasis on their significance in healthcare applications. The paper classifies these sensors based on their operating frequency distinguishing between high-frequency sensors, which operate above 10 MHz, and low-frequency sensors, which operate below this level. The operating principles of breathing sensors as well as the materials and fabrication techniques employed in their design are addressed. The existing research highlights the need for robust and flexible materials to enable the development of reliable and comfortable sensors. Finally, the paper presents potential research directions and proposes research challenges in the field of flexible and wearable respiration sensors. By identifying emerging trends and gaps in knowledge, this review can encourage further advancements and innovation in the rapidly evolving domain of flexible and wearable sensors.

## 1. Introduction

Wearable sensing is an emergent technology for the monitoring of human vital signs in various fields such as health, sports, and the military [1,2,3]. Nowadays, these sensors have gained widespread usage for measuring vital signs in remote health care. Such sensors provide an opportunity to analyze the subject’s health at their own premises, significantly improving the possibility of adaptive medication. Consequently, this has greatly reduced the cost linked with conventional healthcare facilities [4]. Furthermore, the integration of these sensors into textile fiber and clothes for direct connectivity with the human body is employed for health monitoring [5]. Such flexible wearable sensors are able to measure several vital signs, including body temperature, blood pressure, heart rate, breathing rate, etc. [6,7,8]. While many flexible wearable respiration sensors are in their early stages of development, there are already commercially available devices such as Hexoskin and the Zephyr Bioharness etc. [9]. However, these systems are currently lacking in terms of robust analysis software, comprehensive validation studies, and standardized testing protocols.

Among the vital signs, the breathing rate holds particular significance in human physiology, as it contains a wealth of information about a person’s health condition. The normal respiratory rate for a healthy adult typically falls within the range of 12–20 breaths per minute (bpm). However, in specific situations such as high-intensity exercise, the respiratory rate may even increase to as much as 60 bpm [10]. Therefore, monitoring the abnormal respiratory rate (<6 bpm or >24 bpm) is a better predictor of mortality than the heart rate or hypertension. Generally, patients are observed physically to estimate their breathing rate by counting the chest expansion and contraction for a short period of time, which may result in false conclusions about the patient’s health [11]. Therefore, sensors that can monitor breathing in real-time have the potential to provide important diagnostic and monitoring tools for respiratory diseases such as asthma, chronic obstructive pulmonary disease (COPD), and sleep apnea [12].

Furthermore, breathing rate is an important predictor for cardiac arrest, as reported in clinical studies [13,14]. Likewise, monitoring of the breathing rate is helpful for early diagnosis of respiratory illnesses such as COVID-19. In context, an estimation algorithm has been established for the detection of coronavirus owing to a correlation between abnormal breathing rate and COVID-19 infection [15]. Moreover, respiration sensors have emerged as a valuable tool for monitoring infant health in real time. These sensors facilitate the continuous assessment of respiratory patterns, enabling timely identification of irregularities and facilitating prompt medical intervention [16,17].

Several approaches that employ sensors to measure breathing rate for respiratory monitoring exist in the literature. These approaches vary on the basis of sensing techniques, sensor types, and sensor placements, among other factors. The literature presents a prevalent categorization into two main types, namely, contact and contactless sensors. As the name implies, contact-based sensors require physical contact with the subject, while contactless sensors measure breath parameters without physical contact using optical or electromagnetic principles, detecting variations in light or electromagnetic waves caused by breathing. A comprehensive overview of various sensing techniques for both contact and non-contact methods has been presented by [18,19]. Contact-based methods encompassed acoustic sensing, airflow detection, chest and abdominal movement measurement, transcutaneous CO_2_ monitoring, pulse oximetry (SpO_2_), and electrocardiogram-derived methods, while non-contact methods mainly included radar-based detection, optical techniques, ultrasound sensors, and thermal imaging.

Massaroni et al. [20] has conducted another comprehensive review of contact-based respiratory rate sensors. This study identified seven contact-based techniques by monitoring the respiratory airflow, respiratory sounds, air temperature, air humidity, air components, chest wall movements, and modulation of cardiac activity. Moreover, various sensor types were highlighted, including flowmeters, anemometers, fiber optic sensors, microphones, thermistors, thermocouples, and a wide variety of other sensors including pyroelectric, capacitive, resistive, nanocrystal, nanoparticle, infrared, inductive, transthoracic, inertial, ECG, and PPG sensors, among others. While contact-based sensors offer greater precision, they can be uncomfortable and potentially impact the breathing patterns of users. Consequently, wearable sensors have emerged for continuous and seamless breath monitoring. Vanegas et al. [21] recently conducted a systematic review of respiration sensing systems, revealing that most authors opted for detecting chest wall movements in the wearable category, while temperature and humidity of exhaled/inhaled air were the second and third most commonly employed techniques.

**Figure 1:**
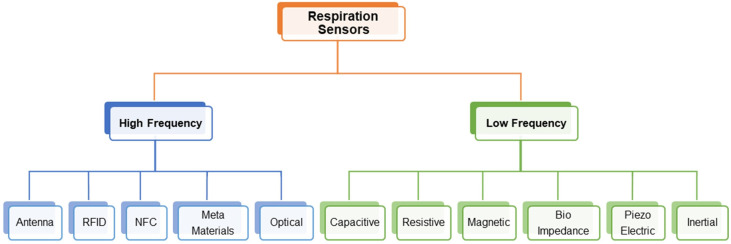
Classification of respiration sensors based on operational frequency.

Furthermore, these breath monitoring techniques utilize a wide range of frequencies, allowing for their classification based on operational frequencies. Thus, flexible wearable sensors for breathing can be broadly categorized into high-frequency systems that operate in the microwave range and relying on changes in electromagnetic properties of surroundings and low-frequency systems that use passive sensing mechanisms to detect breathing motion. This classification is especially useful in the context of Specific Absorption Rate (SAR), which denotes the energy absorbed by human tissue when exposed to electromagnetic radiation. Current international standards mandate SAR testing for frequencies above 4 MHz [22]; however, practical devices that require this testing, such as RFID and NFC-based sensors, typically operate at frequencies of around 13 MHz or above. Therefore, in this review, sensors operating above 10 MHz are classified as high-frequency sensors while those operating below this threshold are categorized as low-frequency sensors, as depicted in Figure 1. This paper aims to provide an overview of flexible and wearable sensors for breath rate monitoring according to the operating frequency, specifically focusing on the popular sensing mechanisms, materials, and fabrication techniques.

For this review, conference proceedings and full-text articles were selected from a broad search containing diverse foundations and catalogs such as Science Direct, Web of Science, MDPI, and IEEE Xplore. Keywords were selected in each source as follows: (Flexible OR textile) AND (wearable AND sensor) AND (respiration OR breathing). The initial search returned 476 results, indicating that a significant number of studies have been conducted in recent years. Consequently, we refined our search for biophysical sensors by placing emphasis on the previously mentioned top three respiration sensing techniques identified in [21]. Then, the results were further analyzed to eliminate duplicates, resulting in a final count of 105 studies. All content of the subsequent sections on the sensing mechanisms, materials, and fabrication techniques are extracted from these studies. The organization of this paper is as follows. Section 2 and Section 3 provide a detailed description of high-frequency and low-frequency respiration sensors. In Section 4, materials and techniques related to respiration sensors are presented and discussed. Finally, Section 5 summarizes the findings of this study and presents recommendations for future research in this field.

## 2. High-Frequency Sensors

High-frequency sensors offer unique advantages such as smaller size and wireless communication capabilities, making them suitable for various applications in healthcare and wearable technology. Among these sensors, antenna sensors, RFID/NFC-based sensors, metamaterial sensors, and Fiber Bragg Grating (FBG) sensors have emerged as the most promising candidates for high-frequency respiration monitoring. In the following subsections, a brief overview of each technique is presented, including its operating principle and associated challenges.

### 2.1. Antenna Sensors

Antenna sensors have become increasingly prominent in the last few years for the monitoring of vital signs, especially for respiration. The sensing mechanism is primarily based on two factors: chest wall movement, and displacement of air volume in the lungs. The movement of the chest wall leads to changes in the physical dimensions of the antenna, while the displacement of air volume alters the dielectric properties in the vicinity of the antenna. Both of these effects result in the variation of electrical characteristics of the antenna, such as its reflection coefficient, resonance frequency, and radiation pattern [23]. The variations in the reflection coefficient (S11) of the antenna sensor caused by breathing can be measured through a vector network analyzer (VNA) and then processed for estimation of the breath rate and pattern recognition. Of course, this scheme is limited to the laboratory and valid only as a proof of concept. Another more practical method is based on the utilization of a tuned frequency transmitter connected to an antenna sensor and monitoring of the backscatter power intensity or received signal strength indicator (RSSI) at the receiver end. The RSSI varies, as the impedance of antenna changes during breathing; therefore, the effective transmitted power varies accordingly.

**Figure 2:**
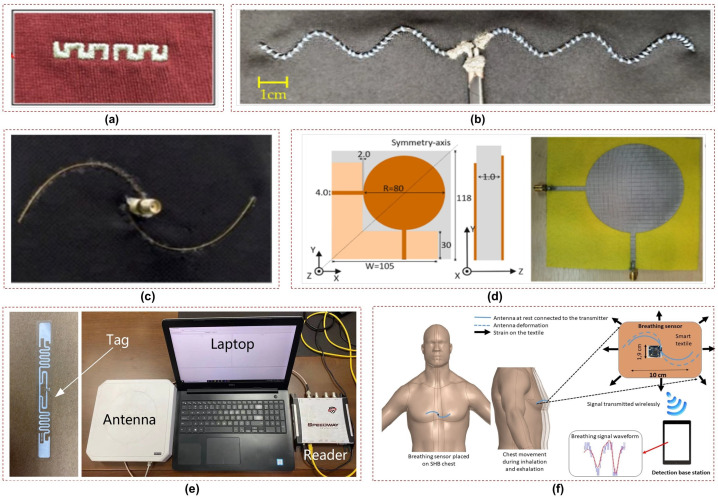
Antenna–based respiration sensors utilizing: (**a**) meandering dipole antenna [24]; (**b**) sinusoidal dipole antenna [25]; (**c**) spiral dipole antenna [26]; (**d**) circular patch antenna [27]; (**e**) RFID–based respiration system [29]; (**f**) Bluetooth–based respiration system [31].

Various types of flexible and wearable antenna-based respiration sensors have been developed for respiration monitoring. For instance, an embroidered meandering dipole antenna-based sensor was integrated into a commercially available t-shirt that detects resonance frequency shifts induced by chest movements during breathing [24], as shown in Figure 2a. In another study, a wearable strain sensor based on a flexible and sinusoidal dipole antenna was fabricated utilizing a conductive polymer material [25], illustrated in Figure 2b. Similarly, flexible and spiral dipole antennas have been designed using multi-material fibers for respiration monitoring [26], as shown in Figure 2c. Moreover, a low-profile fully textile antenna was used as a passive e-textile respiration sensor that relied on the interaction between the human body and the antenna to detect changes in dielectric properties induced by breathing in [27]. This antenna was implemented using a conductive fabric on a felt fabric substrate, providing simplicity and compatibility with different textiles, as shown in Figure 2d.

Commercial RF platforms such as RFID and Bluetooth have been employed for wireless sensing by utilizing the variations in the received signal strength indicator (RSSI). For instance, Patron et al. presented a sensor that employed knitted antennas and inductively coupled RFID tags for comfortable and battery-free monitoring of respiration patterns [28]. Figure 2e depicts a complete RFID-based system for the simultaneous respiration monitoring of multiple persons [29]. Likewise, a number of portable wireless communication platforms were presented in [30,31] that integrate antennas with Bluetooth modules in wearable t-shirts, as shown in Figure 2f. This enables non-invasive and contactless breath detection, with potential applications in medical diagnostics and respiratory condition monitoring.

Nevertheless, the development of flexible and wearable antenna sensors is accompanied by several significant challenges. First, achieving conformity to complex surfaces such as the human body poses a key hurdle. These antennas must be able to adapt and conform to irregular and curved surfaces while maintaining reliable signal reception and transmission. Second, balancing performance and efficiency in a compact and flexible form factor is crucial, as the human body acts as a very lossy medium that absorbs and attenuates electromagnetic signals. This results in decreased antenna efficiency, reduced signal transmission range, and altered radiation patterns. Therefore, wearable antennas need to exhibit high radiation efficiency, wide bandwidth, and reliable communication capabilities by carefully tackling these challenges.

In addition, the safety of subjects is a crucial aspect to consider in the application of antenna sensors. Compliance with specific absorption rate (SAR) limits is essential to ensure the well-being of users. It can be observed from Figure 2 that dipole antennas are employed mostly for sensing in the UHF and ISM frequency bands due to their simple geometry and ease of fabrication. However, these antennas radiate a substantial amount of power toward the subject; thus, further investigation into other antenna types that have better safety features for the end users is required.

### 2.2. RFID/NFC Sensors

Wireless transmission of sensor data can be achieved through various technologies, including radio-frequency identification (RFID), near-field communication (NFC), and Bluetooth. However, when comparing RFID/NFC technology to Bluetooth one notable advantage is the potential for battery-free communication. The principle of operation for RFID is backscattering, where the tag receives energy from the reader’s transmission and uses it to send back a reply. In this case, the sensors can derive power from the electromagnetic (EM) field generated by a remote reader.

#### 2.2.1. RFID Sensors

With the advent of the Internet of Things (IoT) era, radio-frequency identification devices (RFID) sensors have gained popularity for wearable sensing. In particularly, passive UHF RFID sensors have become used widely due to their small size and lack of power requirements. These sensors have the advantage of being lightweight and comfortable due to their flexible nature. Typically, RFID systems are utilized for sensing applications in two manners. The first involves the measurement of back-scattered power intensity, while the second entails the transmission of sensor data towards the reader.

For respiration measurements, RFID sensors have been mostly used as strain sensors utilizing the principle of backscattering. In this case, the main sensing element is an antenna which is specially designed according to the impedance of RFID chip and fabricated on a flexible or textile material. When a strain force is applied to an RFID tag, the physical deformation of the embedded antenna causes a shift in its resonant frequency. This results in a variation in the backscattered power (RSSI) transmitted from the passive RFID tag, which can be used as a metric for detecting mechanical deformations. Thus, RFID-based respiration sensors are designed by correlating the RSSI variation to the mechanical deformation of the RFID antenna. This approach has been demonstrated in various studies for monitoring respiration rate and detecting breathing patterns [16,28,32,33], as discussed in the previous section.

Apart from RSSI, RFID technology is utilized to transmit data from other sensors towards the reader. In a study on graphene-based respiration sensing, the AMS SL900A microchip was employed for this purpose [34]. The SL900A is an Electronic Product Code (EPC) tag that operates in both semi-passive mode (battery-assisted passive) and fully passive mode (without battery) and exhibits power sensitivities of −6.9 dBmW and −15 dBmW in these respective power modes. The EPC chip features a direct connection to the antenna along with a 10-bit analog-to-digital converter (ADC) capable of controlling two external analog sensors. Additionally, it incorporates an integrated temperature sensor with a programmable dynamic range of −40 to 150 °C. This demonstrates that a wearable RFID system with such capabilities enables simultaneous multi-parameter sensing, specifically for humidity and temperature measurements, for subsequent respiration monitoring.

#### 2.2.2. NFC Sensors

NFC is a specific subset of RFID technology that is designed for short-range wireless systems. One significant advantage of NFC over conventional RFID is its ability to facilitate peer-to-peer communication between an NFC-based system and any NFC-enabled smartphone acting as a remote reader. This feature makes NFC technology accessible to individual users and thus expands its potential applications. Similar to RFID, the backscattering technique can be applied to NFC for sensing purposes as well.

An embroidered inductive strain sensor was introduced in [35] for respiration sensing that comprises two embroidered planar coils connected in series, with their mutual inductance being influenced by their relative positions. These coils can serve as antennas for NFC tags that modulate the carrier wave from the reader according to the inductance variations of the sensor, enabling wireless extraction of sensing information. Moreover, an NFC-based smart bandage incorporating wireless strain and temperature sensors is presented in [36]. The bandage utilizes a battery-free NFC transponder (RF430FRL152H) as an intermediary between the sensors and a smartphone application. This enables the acquisition and transmission of data from both sensors. The smartphone application can power the system and provide real-time data acquisition from the sensors at a distance of 25 mm. The authors proposed potential healthcare applications for the smart bandage, including the assessment of respiratory diseases through its usage as a wearable strain sensor.

Despite the inherent flexibility and lack of power requirements of passive RFID tags, a limitation of these tags is the inclusion of an electronic chip. This limits their large-scale production for wearable applications and renders them susceptible to harsh environmental conditions. Therefore, the future of RFID wearable sensing lies in chipless technology, which is more robust and feasible for production due to the absence of any electronic component [37].

### 2.3. Metamaterial Sensors

Electromagnetic metamaterials are man-made materials comprised of structures with electromagnetic properties that are deliberately engineered to offer a range of responses that are difficult or impossible to achieve in naturally occurring materials or composites. The growing popularity of metamaterials in sensing applications is evident from the increasing number of published works utilizing these engineered structures [38]. In these applications, changes in the resonance frequency or amplitude at resonance are typically used to detect variations in the measured parameter. However, it is worth noting that only a small number of studies in the existing literature have employed metamaterials to design flexible and wearable sensors for respiration monitoring.

#### 2.3.1. Frequency Selective Surfaces

**Figure 3:**
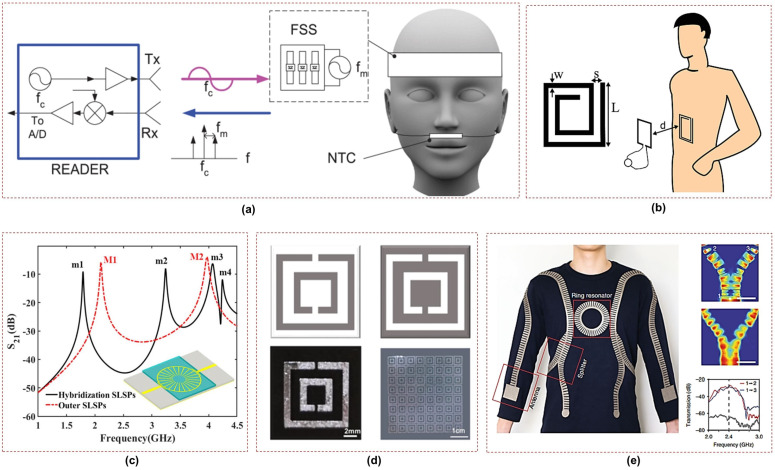
Metamaterial–based sensors: (**a**) modulated FSS respiratory sensor, reprinted with permission from ref. [40]. 2017, IEEE; (**b**) spiral resonator tag respiratory sensor, reprinted with permission from ref. [41]. 2022, IEEE; (**c**) typical surface plasmon resonator sensor [42]; (**d**) liquid metal–based flexible FSS [43]; (**e**) textile–based flexible metamaterial, reprinted with permission from ref. [44]. 2019, Springer Nature.

Frequency Selective Surfaces (FSS) are two-dimensional planar structures consisting of periodic arrays of sub-wavelength elements. They are designed to selectively transmit or reflect electromagnetic waves based on their frequency. FSSs are widely utilized in microwave frequency filters, radar absorbing materials (RAMs), and antenna reflectors [39]. For respiration sensing, a wireless apnea detector was proposed in [40] that utilizes a passive respiration sensor to measure the changes in airflow temperature during breathing, as shown in Figure 3a. A transponder based on a modulated FSS is employed that uses a backscattered field technique for sensing and is composed of an array of dipoles loaded with varactor diodes. The resistance of thermal sensor changes due to the variations in airflow temperature during breathing which modulates the backscatter response. An algorithm based on peak detection has been used to calculate real-time respiration and apnea intervals. However, this system requires a battery to polarize the FSS, and it is rather uncomfortable for the patient due to the many interconnected devices worn on the face and head.

Traditional frequency selective surfaces used for sensing present certain limitations in terms of flexibility, as the materials and structures used are rigid. Therefore, liquid metal-based technologies [43] and textile-based substrates [44] are feasible options that can be explored for designing flexible metamaterials for wearable applications, as depicted in Figure 3d,e.

#### 2.3.2. Spiral Resonator Tag

Planar spiral structures (SRs) are commonly used as lumped inductors in microwave circuits and serve as a fundamental element for metamaterials [45]. The inductance of a spiral is determined by its geometry (such as square, circular, or polygonal), number of turns, turn width, and spacing between turns. A breath rate sensor based on an SR tag is illustrated in Figure 3b; it is printed on a thin, flexible textile substrate suitable for wearable applications [41]. The sensor detects the respiratory movement of the abdomen during inspiration and expiration. A microstrip probing loop serves as the reader antenna and measures the variation in the real part of input impedance caused by the change in the normal distance between the probing loop and the SR tag. Thus, the respiratory rate is obtained by analyzing the amplitude-modulated signal captured by the probe. The sensor was employed to experimentally measure the breath rate of a test subject in a quasi-real scenario. To enhance the capabilities of the presented sensor, however, future advancements should focus on transitioning from quasi-real environments to a fully operational settings by integrating a signal acquisition board.

#### 2.3.3. Surface Plasmons Resonators

Spoof localized surface plasmons (LSPs) are electromagnetic modes that are highly confined and can be realized using various techniques such as textured metal surfaces, thin metal layers, and conductive textiles [44,46,47]. These modes can be used to design wearable sensors by detecting changes in localized electromagnetic fields. Although these modes are associated with strong subwavelength confinement, which is a characteristic feature of LSP resonances, they extend into the surrounding space evanescently, enabling interactions for sensing and mode excitation. Figure 3c shows the structure of a typical spoof LSP resonance sensor with a patterned conductive structure.

A wearable textile sensor based on LSP resonance was proposed in [48] for vital sign monitoring such as breathing and heartbeat. The sensor is placed on the chest and excited remotely through a curved dipole antenna. Small movements of the chest during breathing and heartbeat change the resonant frequency of the sensor, which is remotely monitored from the excitation source. Moreover, an energy-efficient and secure wireless body sensor network (WBSN) interconnected through radio surface plasmons on metamaterial textiles was introduced [44], as illustrated in Figure 3e. This conductive fabric shows a three-fold improvement in transmission efficiency compared to conventional radio networks. Wireless communications remain confined to within 10 cm of the body, ensuring greater privacy and security. This textile can be used in conjunction with a range of sensors, including respiration sensors, for the monitoring of vital signs.

Resonators relying on surface plasmons generally have a very high quality factor, as the electrical field is confined in a very limited space. However, these sensors face challenges in terms of the complexity of their fabrication and sensitivity to the environment. Therefore, further research is needed to ensure their reliable performance in the context of wearable sensing.

### 2.4. Fiber Bragg Gratings

**Figure 4:**
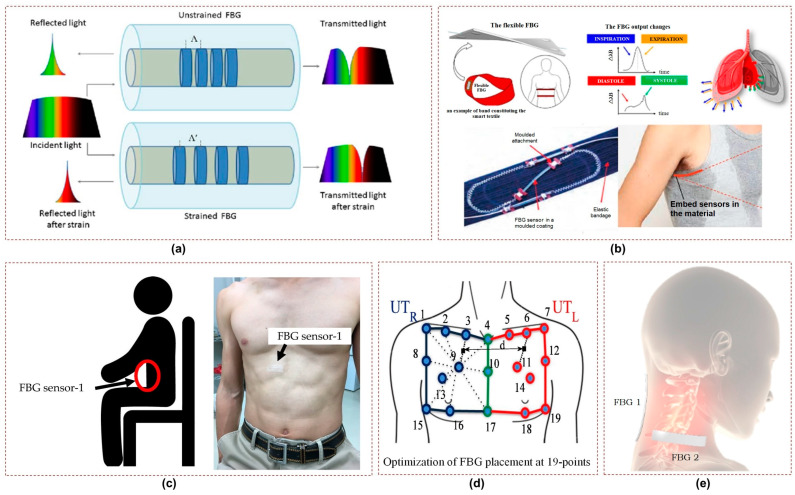
Fiber Bragg Grating (FBG) sensors: (**a**) working principle of FBG [49]; (**b**) application in vital signs monitoring [50]; (**c**) single FBG respiratory sensor [51]; (**d**) multiple FBG respiratory sensors [52]; (**e**) monitoring neck movement and respiration [53].

Fiber Bragg Grating (FBG) sensors are well suited for measuring biophysical parameters, including the respiratory behavior of the chest and abdominal regions. These sensors are comprised of a fiber optic grating that reflects a specific wavelength of light [49], as shown in Figure 4a. When the FBG undergoes strain or displacement caused by breathing, its reflected wavelength experiences a corresponding shift. This shift is directly proportional to the applied strain or displacement, enabling FBG sensors to accurately measure and monitor breathing patterns. Various applications of FBG sensors for the monitoring of vital signs are illustrated in Figure 4b, including cardiac and respiration activities [50]. Koyama et. al demonstrated in a study that FBG sensors can efficiently detect respiratory strain in the abdomen, chest, and shoulder regions [51], as shown in Figure 4c.

FBG sensors are especially advantageous during MRI testing thanks to their immunity to strong magnetic fields. A study focusing on the design and assessment of an MR-compatible smart textile utilized six FBGs [54]. The objective was to non-intrusively monitor and characterize a patient’s respiration. The study employed the chest wall kinematics during breathing to optimize the positioning of FBGs on the textile and improve their sensitivity in monitoring compartmental volume changes. Figure 4d illustrates a similar study in which the positioning of FBGs on the textile was determined using an optoelectronic system [52]. Another study evaluated an MR-compatible smart textile with FBG sensors for monitoring respiratory and cardiac activities during apnea and quiet breathing [55]. The results showed that accurate measurement of respiratory parameters and heart rate could be obtained without image artifacts in the MR environment.

Furthermore, elastic belts integrated with optical fibers for monitoring breathing activities were studied in [56]. The aim was to assess the impact of differences in the participants’ positions on the breathing patterns detected by the optical fibers. Experiments were conducted using FBG sensor arrays to monitor breathing patterns. Two sets of FBG arrays, each comprising five sensors, were placed at specific locations on the body, namely, the abdomen and chest. The diversity technique was employed to enhance detection accuracy. The results showed consistent accuracy for individuals, though variations were observed among the participants. In the same year, a wearable system was proposed by utilizing two FBG sensors positioned on the neck to monitor neck movements and breathing [53], as depicted in Figure 4e. This system effectively captured flexion extension, axial rotation, and respiratory frequency parameters, showing comparable performance to optical reference systems. The estimated mean and breath-by-breath respiratory frequency values exhibited errors ≤6.09% and ≤1.90% during quiet breathing and tachypnea, respectively, demonstrating high accuracy. Additionally, a 3D printed sensor using FBG technology was introduced for respiratory and heart rate monitoring in [57]. Each sensor was equipped with a single FBG fully encapsulated within a 3D-printable flexible material during printing.

The biggest challenge associated with FBG sensors is that the optical fibers are typically rigid and fragile. Therefore, they need to be encapsulated in a material that can provide protection, flexibility, and biocompatibility in order to make them suitable for flexible and wearable applications. Such encapsulation additionally shields the FBG sensor from external influences such as moisture, mechanical stress, or bending which could compromise its performance or usability.

## 3. Low-Frequency Sensors

Various low-frequency sensors have been developed to enable non-invasive and continuous breath monitoring. These sensors are often characterized as simple and passive devices that typically operate at sub-megahertz frequencies and do not require an external power source for their operation. They are relatively straightforward in terms of their design and implementation, often consisting of basic lumped component designs such as capacitors, resistors, coils, or electrodes. The following section discusses the most commonly used low-frequency techniques for flexible and wearable respiration sensors.

### 3.1. Capacitive Sensors

Capacitive sensors are a type of flexible wearable sensor that has shown promising scope for monitoring breathing and respiration. The working principle of capacitive sensors relies on variations in capacitance due to changes in electrode dimensions, the separation distance between electrodes, or dielectric properties. These changes allow the capacitive sensor to detect physical quantities such as strain, pressure, or humidity. Capacitive sensors on the human body have been utilized in various ways to detect breathing. They can measure chest expansion and contraction during respiration by detecting changes in capacitance; additionally, these sensors can be employed to detect air humidity from exhaled breaths as a means of monitoring respiration. Another approach is to measure the pressure on the abdomen and chest during exhalation, which can be used to monitor respiratory movements.

**Figure 5:**
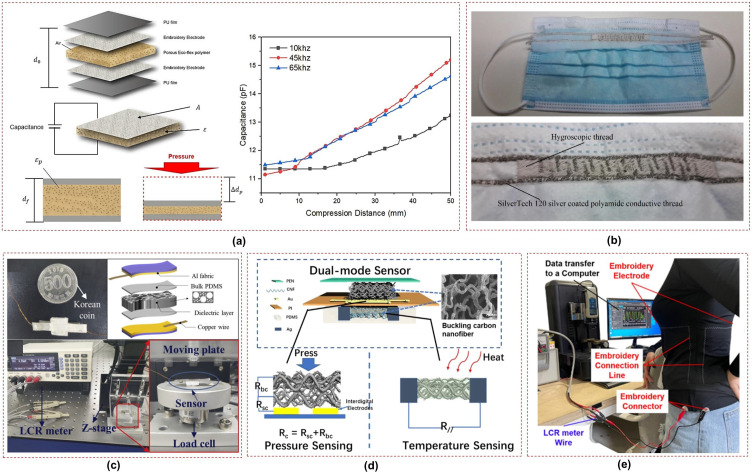
Capacitive sensors utilizing: (**a**) porous Ecoflex polymer [60]; (**b**) embroidery on face mask [61]; (**c**) PDMS composites, reprinted with permission from ref. [64]. 2021, Elsevier; (**d**) carbon nanofibers [65]; (**e**) embroidered electrodes [66].

The simplicity of the working principle of capacitive sensors has led researchers to explore various fabrication techniques and materials to improve their flexibility and sensitivity for wearable applications. In [58], a flexible capacitive sensor with high sensitivity, a large sensing range, and good stability was developed. This sensor utilizes a micro-structured polydimethylsiloxane (PDMS) film embedded with multi-walled carbon nanotubes as the conductive electrode and a smooth PDMS film as the dielectric layer. The sensor was successfully applied to monitor arterial pulse waves and breathing. In another work, the design of highly sensitive capacitive sensor for wearable sensing applications was reported; this sensor uses conductive textile electrodes and polyurethane (PU) foams as the dielectric layer. Unlike previous works that involved complex fabrication processes, this sensor employs a simple and cost-effective technique, with a microporous PU foam serving as the dielectric material [59]. A compression sensor is illustrated in Figure 5a, where the porous Ecoflex dielectric is placed between embroidered electrodes and changes in capacitance were measured to assess respiration [60].

Furthermore, a facemask with an inter-digital embroidered capacitor was proposed for respiration monitoring in [61], as shown in Figure 5b. The sensor operates by exploiting the humidity of exhaled air to monitor respiration. A paper-based wearable screen printed sensor was proposed in [62] for respiration monitoring, particularly for patients affected by SARS-CoV-2. The sensor utilizes traditional screen printing methods to fabricate interdigitated electrodes composed of multi-walled carbon nanotubes (MWCNTs) and polydimethylsiloxane (PDMS) composite on a paper substrate. The paper substrate functions as a humidity-sensitive material due to its hygroscopic properties. As a result, the capacitance of the sensor varies in response to changes in the dielectric constant during inhalation and exhalation. In another study, a flexible paper-based capacitive pressure sensor was fabricated using common daily materials such as paper, polyester conductive tape, and polyimide tape [10]. This sensor has a wide pressure detection range and was able to demonstrate successful application in various scenarios, including nasal respiration analysis. In another study, a capacitive sensor was made using a co-doped barium titanate dielectric material and palladium–silver electrodes [63]. In addition, the screen-printing and micro-dispensing construction methods were evaluated. The resulting sensors demonstrated suitable characteristics for respiration monitoring, with the screen-printed sensors ultimately chosen for experimentation and prototyping due to their greater dynamic range.

Similarly, a sensor for wearable electronics was fabricated in [64] using hierarchically porous PDMS composites with a simple and cost-effective method of screen printing, as shown in Figure 5c. The resulting sensor exhibits a wide pressure measurement range, high sensitivity, and flexibility, making it suitable for various applications in wearable electronics, including respiration. Furthermore, a smart face mask with a dual-sensing mode respiration sensor was presented for recognizing multiple respiration patterns in [65]. By incorporating three-dimensional carbon nanofiber mats, this sensor enables simultaneous pressure and temperature sensing, allowing for real-time recognition and accurate monitoring of up to eight respiration patterns, as depicted in Figure 5d. This mask has potential applications in health monitoring and early detection of breathing-related diseases. Moreover, a fabrication strategy for a multi-functional e-textile based on polypyrrole (PPy) and rose-like silver flower-decorated knitted cotton/spandex fabric (KCSF) is presented in [67]. This e-textile was then used in the construction of a capacitive sensor with an ultra-wide working pressure range and high sensitivity. In addition, Ecoflex rubber has been effectively used as a dielectric in capacitive sensors for respiratory measurements. Wearable capacitive pressure sensors have been developed for human activity and respiration monitoring, with Ecoflex and silver nanowires respectively serving as the dielectric material and electrodes [68,69].

Kang et al. developed a respiration sensor utilizing a variable parallel-plate capacitive design and incorporating nonwoven fabrics [70]. This sensor comprises both stretchable and non-stretchable fabric segments that respond to respiratory movements, leading to alterations in sensor length. In [71], a capacitive sensor was developed based on textiles designed to be worn around the chest as a belt for monitoring the respiration rate. This sensor utilizes the change in capacitance resulting from the expansion of the fabric during respiration, achieving a high precision level in the micrometer range. A zinc ion hybrid supercapacitor (ZHS) with a double-crosslinked hydrogel electrolyte was developed in [72], serving as both an energy storage device and a self-powered sensor for human movement and breathing detection. In [66], a respiration sensor was proposed based on a parallel capacitor embroidered on textiles. This respiration capacitor was characterized using a silver thread and a porous Ecoflex material to simulate air from the lungs. The study then examined actual breathing patterns, including normal breathing, deep breathing, hyperventilation, and apnea, using a clothing-type respiration sensor, as shown in Figure 5e.

Conversely, capacitive sensors have a number of limitations on their use in respiration sensing. One such limitation is their sensitivity to environmental factors such as humidity and temperature, which can affect the accuracy of the measurements. Additionally, the placement of electrodes on the textile substrate may introduce variability in the signal due to movement or displacement during breathing. Therefore, calibration and compensation techniques are necessary to account for these variations.

### 3.2. Resistive Sensors

**Figure 6:**
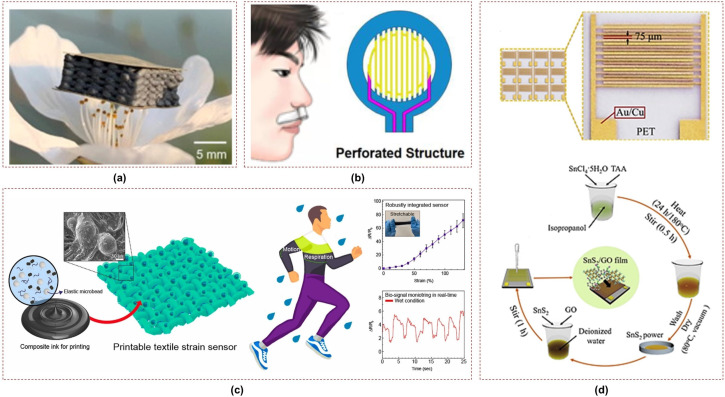
Resistive sensors utilizing: (**a**) graphene–based porous structure, reprinted with permission from ref. [78]. 2022, Elsevier; (**b**) platinum–based perforated structure [79]; (**c**) conductive printing on textiles, reprinted with permission from ref. [80]. 2021, Elsevier; (**d**) tin disulfide/graphene oxide nanoflower on PET, reprinted with permission from ref. [87]. 2019, Elsevier.

Resistive sensors for respiration monitoring employ different sensing mechanisms, including strain sensing, temperature sensing, and humidity sensing. Strain sensing involves flexible materials that changes their resistance in response to respiratory movements, thereby enabling measurement of the respiratory volume or rate. Most strain sensors employ resistive techniques due to the direct proportionality between the resistance of conductive materials and the strain. For instance, a respiration sensor based on stretchable conducting fabric was developed in [73] to detect changes in electrical resistance corresponding to tensile strain during inhalation and exhalation. Moreover, several hydrogel-based flexible strain sensors with self-healing capabilities and high sensitivity have been proposed for human motion and respiration monitoring [74,75]. Textile strain sensors have been designed for conductive yarns by combining them with elastic yarns or making them elastic by coating them with carbon-filled silicon [76,77]. Flexible dual parameter sensors with hierarchical porous structures have been introduced to enhance the functionality of wearable devices for fully decoupled pressure and temperature sensing [78], as shown in Figure 6a. A flexible and perforated nasal-based respiratory sensing system was introduced in [79] and was found to have improved air convection efficiency and thermal sensitivity, as shown in Figure 6b. Platinum was selected as the sensing material due to its stable physical and chemical properties. Moreover, its resistance–temperature characteristics exhibit excellent linearity and rapid thermal response. A printable wet-resistive textile strain sensor was introduced in [80] and demonstrated high sensitivity and mechanical durability for robust wearable electronics, as depicted in Figure 6c. This composite strain sensor was successfully printed onto a tracksuit to enable real-time monitoring of respiration and arm motion signals.

Various flexible humidity sensors based on different materials have been explored for vital sign monitoring by measuring impedance. Highly sensitive textile-based humidity sensors employing graphene oxide/Polypyrrole composites have been developed for real-time respiration monitoring [81,82]. In particular, the field of humidity sensing has seen advancements, with printed flexible humidity sensors utilizing cellulose nanofiber-based composites [83]. A flexible humidity sensor based on a poly-3D hollow fiber membrane was proposed in [84] and was found to exhibit high sensitivity, selectivity, and stability for monitoring physiological activity. In [85], a graphene oxide-based breath sensor was demonstrated to have high sensitivity to humidity and potential applications in detecting lung cancer and sleep apnea. Another study focused on a Coolmax/graphene-oxide functionalized textile humidity sensor with ultrafast response designed for monitoring human activities [86]. In [87], an ultrasensitive humidity sensing capability was achieved using a tin disulfide/graphene oxide nanoflower composite with ultrafast response, low hysteresis, and good reversibility, making it promising for wearable applications, as shown in Figure 6d. Additionally, few-layer NiPS3 and ZIS nanosheet-based flexible humidity sensors have been developed that offer high selectivity along with rapid response and recovery times for real-time monitoring of respiration and environmental conditions [88,89]. Several other flexible and wearable resistive sensors have been designed employing composites of polymers, carbon, bi-metallic nanoalloys, and thermoplastics [90,91,92,93].

Despite the potential advantages, resistive sensors are susceptible to drift and hysteresis, leading to potential inaccuracies in measurements. Moreover, unintended fluctuations in electrical resistance may arise as a consequence of variations in the ambient temperature and humidity conditions. Therefore, signal processing techniques are necessary in order to ensure accurate respiratory monitoring, address noise, and extract relevant respiratory information.

### 3.3. Magnetic Induction Sensors

Magnetic Induction (MI) is a non-contact technique used to measure changes in the impedance distribution of an object. It relies on the electromagnetic coupling between a coil and an object in close proximity. Human tissue is characterized as an electrical medium that is both inhomogeneous and anisotropic in nature. The impedance distribution within the thorax is influenced by physiological activities such as breathing, which can be attributed to various factors, including volume changes and displacement of organ boundaries. Currently, three primary categories of flexible inductive sensors are extensively employed: eddy current sensors, mutual-inductive sensors, and self-inductive sensors.

**Figure 7:**
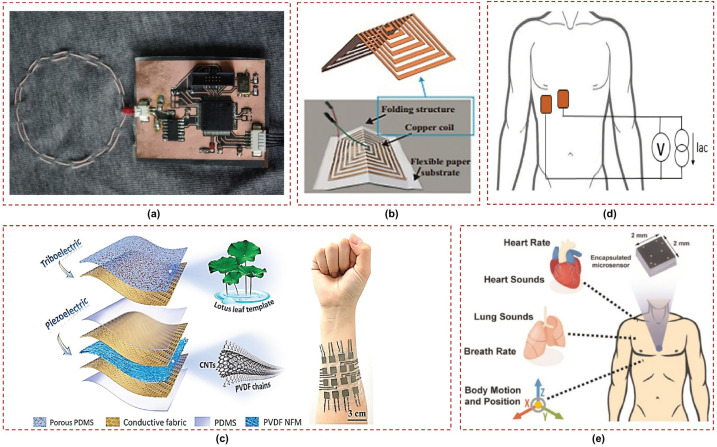
Miscellaneous low–frequency sensors: (**a**) mutual inductive strain sensor [94]; (**b**) self–inductive displacement sensor [96]; (**c**) piezoelectric multi–modal sensor, reprinted with permission from ref. [101]. 2021, Elsevier; (**d**) bioimpedance sensor [105]; (**e**) inertial sensors [106].

In the eddy current method, the coil is driven by an alternating current, generating a primary alternating magnetic field that penetrates the conductive medium surrounding it, thereby inducing eddy currents within the medium. These eddy currents in turn generate a secondary alternating magnetic field that interacts with the primary field, resulting in changes in the coil’s impedance. These changes are directly related to the impedance distribution of the object. When the coil is positioned in front of the thorax, the thorax acts as the object under test and the coil’s impedance varies in response to the variation in thoracic impedance distribution caused by the movement of the lungs. In [94], a system was introduced for long-term monitoring of respiration and pulse. The system utilizes four non-contact sensors based on magnetic eddy current induction; these are integrated into a shirt, as shown in Figure 7a. The effectiveness of this system in monitoring respiration and pulse was demonstrated through measurements conducted on healthy volunteers. Similarly, a flexible device for non-contact monitoring of respiration and pulse was presented in [95]. It combines magnetic induction for respiratory monitoring and reflective photoplethysmography (rPPG) for pulse detection, enabling enhanced signal coverage. Sensor signals are processed by a microcontroller and wirelessly transmitted via Bluetooth to a display unit such as a personal computer or Android device. The device can easily be placed in a shirt or jacket pocket to monitor vital signs.

In addition, inductive sensors can measure physical quantities such as displacement and pressure with high sensitivity utilizing the principles of mutual inductance. Inductive-based displacement sensors utilize electromagnetic induction to measure and monitor object displacement or position. These sensors generate an alternating magnetic field using a coil; the movement of the object induces a change in the magnetic field, allowing for displacement measurement. An embroidered inductive strain sensor was proposed in [35] fabricated using off-the-shelf conductive yarns that can be woven into clothing such as shirts or sweaters. The sensor consists of two embroidered planar coils connected in series, with their mutual inductance affected by their relative positions. The displacement between the coils alters the inductance, enabling the sensor to function as a strain sensor. Moreover, a paper-based self-inductive folding displacement sensor (PSIFS) was proposed in [96]; this sensor operates on the principle of inductance variation caused by the three-dimensional deformation of a planar inductor coil, as depicted in Figure 7b. This research focuses on investigating the structural design principles concerning two key aspects of sensitivity and size. The PSIFS has demonstrated the capability to accurately identify and differentiate various breathing states and motion states of human joints.

Magnetic induction sensors used in respiration monitoring can be susceptible to measurement errors caused by external electromagnetic interference. It is essential to employ calibration and compensation techniques to mitigate variations in the magnetic field arising from different body positions or environmental factors. Furthermore, careful consideration of the sensor’s design and placement relative to the body is crucial to optimize measurement accuracy.

### 3.4. Piezoelectric Sensors

Piezoelectric sensors are fabricated using piezoelectric materials, which exhibit an internal polarization phenomenon when subjected to an external force. This phenomenon leads to the appearance of positive and negative charges on the surfaces of the piezoelectric material, resulting in the generation of a piezoelectric potential. Upon removal of the external force, the polarization phenomenon disappears. Thus, sensors can be designed by using piezoelectric sensors during respiration monitoring to detect variations in the electrical signal.

#### 3.4.1. Piezoelectric Sensors and Generators

Several studies have proposed the use of piezoelectric sensors as part of multi-functional sensing technologies. These studies aim to integrate multiple smart functions into wearable sensors by employing hybrid mechanisms [97,98,99]. In addition, researchers have explored the development of self-powered sensors for portable and wearable devices, aiming to overcome the limitations of operational time, rigid characteristics, and bulky size associated with conventional power sources. For instance, a flexible hybrid nanogenerator was presented in [100] that combines a solar cell, a transparent triboelectric nanogenerator, and a piezoelectric nanogenerator.

Similarly, Zhu et al. designed a self-powered hybrid electronic skin that combines triboelectric and piezoelectric effects to enable wearable multi-sensing [101], as shown in Figure 7c. In [102], a nanogenerator was developed and utilized as a flexible active respiratory sensor, and flexible piezoelectric smart-textile sensor and energy harvester was developed in [103]. This sensor demonstrated its effectiveness and sensitivity in detecting activities such as falling water droplets, footsteps, and breathing. A nasal-based sensor based on a thin film of piezoelectric polyvinylidene fluoride (PVDF) was proposed in [104] for the purpose of monitoring respiratory patterns. The cantilever beam of the sensor is affected by the air exhaled from the nostrils and generates a dynamic voltage signal based on the piezoelectric effect.

#### 3.4.2. Piezoelectric Resonators

Piezoelectric-on-substrate (TPoS) refers to a specific type of piezoelectrically actuated resonators designed with a piezoelectric layer on top of a substrate layer. The incorporation of the substrate layer enhances power handling capabilities and improves the quality factor of the resonator. For respiratory monitoring applications, it is crucial to have a TPoS resonator that exhibits high sensitivity to changes in respiration flow as well as a high electromechanical coupling and quality factor.

A wireless sensor utilizing a thin-film piezoelectric-on-substrate (TPoS) resonator was employed in [107] for respiratory monitoring. Such passive wireless sensors consist of an antenna and a resonator; the resonator detects the respiration profile. The resonator used in this investigation consistsed of a stack of Molybdenum, Aluminum, and Nitride deposited on a diamond substrate. During inhalation and exhalation, TPoS resonators experience two distinct effects. First, the temperature difference between the exhaled breath and the resonator induces a frequency shift due to alterations in the elastic properties of the resonator’s constituent materials. Second, the condensation of water vapor from exhalation onto the resonator’s surface causes a frequency drift. The TPoS resonator is integrated with an RFID tag’s antenna, and respiration monitoring is achieved through backscattering techniques.

The successful integration of piezoelectric materials into wearable sensors requires meticulous design considerations in order to ensure mechanical stability and durability. The accuracy of measurements can be compromised by sensor drift and noise, necessitating the implementation of suitable signal-processing techniques. Calibration and compensation methods are imperative to address individual variations and ensure consistent and reliable measurements.

### 3.5. Bioimpedance Sensors

Bioimpedance sensors for respiration monitoring operate based on the principle of measuring changes in electrical impedance caused by respiratory movements. These sensors typically consist of electrodes that are placed on the skin surface, creating a closed electrical circuit [105], as shown in Figure 7d. When the person breathes, the expansion and contraction of the chest or abdomen lead to changes in the thoracic impedance. These changes in impedance are caused by variations in the conductivity and volume of the underlying tissues and organs. Thus, respiratory patterns can be continuously tracked by measuring these impedance variations.

Impedance sensors play a crucial role in breath monitoring, offering a non-invasive and continuous approach that can track respiratory patterns and gather valuable health information. Several articles have explored the use of such flexible sensors to monitor physiological activities. For instance, a wearable smart clothing system was proposed in [108] integrating numerous sensors for accurate respiratory rate estimation in a home-care monitoring context. Additionally, a portable physiological signal recorder was designed and implemented in [109], achieving a 24 h operating time and focusing on electrocardiography, bioimpedance, and user activity measurements. Moreover, the development of a wearable monitoring system named Wealthy enabled continuous remote monitoring of electrocardiogram and impedance pneumography signals and was able to discriminate between different breathing patterns using piezoresistive fabric sensors [76]. In [110], a textile sensing system was employed for the simultaneous acquisition of electrocardiogram and impedance pneumography signals using fabric sensors and electrodes.

In addition, a wearable scheme incorporating an electrical impedance tomography (EIT) belt has been designed with a novel active electrode architecture, high image frame rate, and wide operating bandwidth, enhancing the neonatal thorax monitoring for vital signs [17]. Moreover, the integration of MEMS stethoscope, ambient noise sensing, ECG, impedance pneumography, and nine-axial actigraphy into a wearable multimodal stethoscope patch in [111] allowed for long-term auscultation and high-quality signal acquisition. Additionally, a high frame rate wearable EIT system utilizing active electrode ASICs was developed in [112], achieving accurate measurement of EIT signals for lung respiration and heart rate monitoring. Lastly, an energy-efficient and reconfigurable sensor IC was implemented in [113] to improve bioimpedance spectroscopy and ECG recording in wearable health devices by enhancing the accuracy of signal measurements.

Bioimpedance measurements are sensitive to electrode placement and contact quality, and require careful attention to ensure consistent and reliable measurements. Calibration procedures and algorithms are necessary in order to accurately interpret impedance changes related to respiration and distinguish them from other physiological signals.

### 3.6. Inertial Sensors

The primary characteristic of respiration involves the movement of air in and out of the lungs, which results from the expansion and contraction of the lungs. The associated ribcage movement can be detected through inertial sensors [106,114], as evident from Figure 7e. These sensors offer versatility and non-obtrusiveness, making them suitable for various applications and providing a reliable and cost-effective method of collecting motion data. A wearable multi-sensor patch was developed for the purpose of detecting and recognizing human breathing patterns in [115]. This multi-sensor patch, composed of an accelerometer and a pressure sensor, was shown to accurately measure breathing-related inertial motion and muscle stretch when attached near the diaphragm. Through analysis of different breathing motions, including inhalation, exhalation, normal breathing, and breath-holding conditions, the breathing frequency and normal breathing rate were determined using an accelerometer and flexible capacitive pressure sensors.

However, inertial measurement units (IMUs) are mostly incorporated to capture motion data and facilitate activity monitoring in conjunction with other sensors. For instance, the NeoWear system was developed to monitor neonatal vital signs using a sensor belt which comprises a pressure sensor and an Inertial Measurement Unit (IMU) [116]. This system detects respiration rate and apnea events by analyzing the pressure and movement data of the respective sensors. The accuracy of inertia-based methods for detecting body movements can be affected by motion artifacts. In the context of respiration monitoring, motion artifacts can undermine the ability to accurately capture and analyze respiratory patterns, potentially leading to erroneous or incomplete results. Therefore, mitigating and minimizing the impact of motion artifacts is a critical consideration when developing and implementing inertial sensors for respiration monitoring.

## 4. Manufacturing Materials and Fabrication Techniques

**Figure 8:**
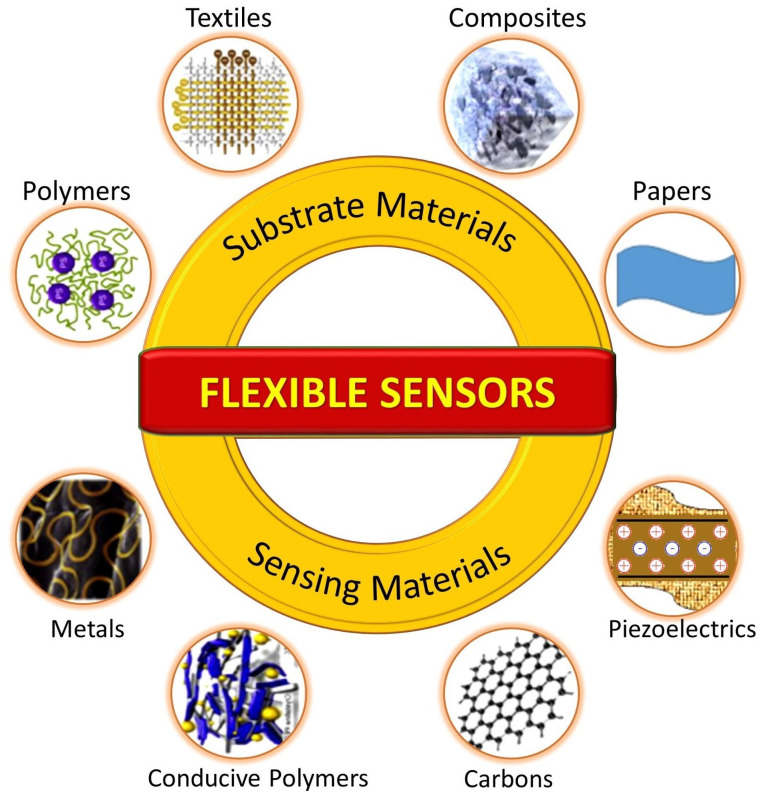
Typical flexible materials for wearable respiration sensors.

Many materials and techniques have been explored to date for the fabrication of flexible wearable sensors. The fundamental requirement for these sensors is to exhibit improved performance when applied to the human body while simultaneously possessing material flexibility, biocompatibility, and user comfort. Sensors are primarily comprised of two components, namely, the substrate and the sensing elements, which are connected by metallic interconnectors. This section provides a brief overview of substrate and sensing materials utilized in the context of respiration sensors, as depicted in Figure 8.

### 4.1. Substrate Materials

Selection of the substrate plays a crucial role in designing wearable sensors, as the substrate is essential for ensuring that the sensor can conform to the body’s contours and withstand its movements while maintaining optimal performance and providing a satisfactory user experience [117].

#### 4.1.1. Polymeric Materials

Polymer-based materials have been extensively used as substrates for flexible sensors. These are heavy-weight molecules composed of repeating units that exhibit distinct characteristics based on their internal bonds. Polymers offer tunable chemical, structural, and electrical properties, making them attractive candidates for sensing applications. Several types of polymer-based materials have been used as substrates for flexible wearable respiration sensors. Polydimethylsiloxane (PDMS) is a silicone-based elastomer known for its excellent flexibility, biocompatibility, and optical transparency, and is the most commonly used substrate material for wearable sensors [58,64]. It has also been used as an encapsulation material for protecting fragile sensors such as FBGs [57]. In addition, Polyurethane (PU) has been used for respiration sensors [59,91], as it is softer than PDMS. Polyimide (PI) is another prevalent substrate material for wearable sensors, which has been used for flexible wearable sensing due to its high tensile strength and flexibility [10,118,119]. Furthermore, rubbers such as Ecoflex are employed for breathing applications, representing a skin-safe substrate material [68,69]. These rubbers have modulus similar to that of human skin, which enhances their adaptability to the human body.

#### 4.1.2. Textiles and Fabrics

Wearable sensor technologies have witnessed significant advancements in utilizing fabric and textile materials as substrates. These materials offer flexibility, durability, and comfort, making them highly suitable for a wide range of applications. Flexible sensors integrated into textile materials offer comfort, wearability, and ease of use. Several articles have focused on the integration of sensors into fabric and textiles to enable real-time monitoring of vital signs and respiration. For instance, wearable antennas and sensor tags are embroidered or printed on fabric substrates, ensuring unobtrusive and comfortable monitoring experiences [24,41]. These materials have been successfully utilized in various applications, such as knitted antennas that utilize wool, lycra, and conductive fabrics to achieve both elasticity and functionality [16,28]. Cotton T-shirts serve as a particularly ideal platform for such applications, providing both comfort and convenience to the subject [25,26,30,31].

In terms of specific sensing techniques, strain sensors have been fabricated on stretchable fabrics woven with fine yarns [80], while pressure sensors have utilized knitted cotton/spandex fabric [67]. Additionally, textile-based sensors have been developed for monitoring respiration through capacitive sensing [66]. Textile-based capacitive sensors can be fabricated using screen-printed silver ink electrodes on non-stretchable nonwoven textiles, providing good flexibility and robustness [71]. Moreover, fabric-based electrodes [108] and metamaterial textiles [44] have been employed to facilitate wearable sensing, and other textile substrates such as nonwoven fabrics [70] and knitted fabrics [76] have been explored for cardio-respiratory monitoring. The utilization of fabrics and textile substrates demonstrates the versatility and potential of integrating sensors and antennas into wearable technologies, enabling the development of comfortable, functional, and personalized smart textiles.

#### 4.1.3. Composite Materials

A variety of composite materials employing different types of materials have been investigated for their applicability in respiration sensors. Thin films of Si/SiO_2_ multilayer composite can serve as the substrate for piezoelectric respiration sensors [34,97]. In another study, co-doped barium titanate was used as the dielectric material for a capacitive respiration sensor, allowing for both miniaturization and flexibility [63]. Furthermore, hydrogel composites that are soft and stretchable materials have been employed for monitoring vital signs [74,75,120]. A zinc-based hydrogel was employed as the dielectric material for the development of a supercapacitor for self-powered sensors in [72]. In addition, a composite material consisting of sodium alginate, polyacrylamide, and gallium was employed for a strain-sensitive hydrogel that could be used in wearable electronic devices [81].

The mechanical and biocompatibility characteristics of commonly used substrate materials are summarized in Table 1.

#### 4.1.4. Paper-Based Materials

Paper-based (PB) sensors have been widely reported for respiratory detection owing to their excellent biocompatibility, cost-effectiveness, and eco-friendly nature. The primary material used in paper-based sensors is cellulose, which is a naturally occurring biopolymer. For this reason, PB sensors generally exhibit good biocompatibility. Furthermore, their lightweight and flexibility ensure ease of use for continuous monitoring without causing significant inconvenience to the wearer. PB sensors are very cost-effective, as the primary raw material is abundant and inexpensive and the manufacturing processes are not highly specialized. This cost-effectiveness makes them easily accessible for a wide range of healthcare applications. Another important feature is that paper sensors are biodegradable, meaning that they can naturally break down over time without leaving behind harmful residues. Therefore, environmentally friendly and disposable sensors can be fabricated with paper-based materials at a very fractional cost.

In addition, the PB sensors can be easily customized and adapted to various respiratory parameters, including humidity and pressure. For instance, a noninvasive wearable screen-printed sensor was proposed in [62] for human respiration monitoring using humidity sensing technique. The proposed sensor utilizes multi-walled carbon nanotubes (MWCNTs) and polydimethylsiloxane (PDMS) composites on a paper substrate. During inhalation and exhalation, the hygroscopic nature of paper causes a change in the dielectric constant, which in turn changes the capacitance of the sensor. Moreover, a low-cost approach was proposed to fabricate a flexible PB capacitive pressure sensor using common materials in [10], where 80 g/m^2^ printing paper was used as the dielectric material and polyester conductive tape as flexible electrodes. Likewise, a paper-based self-inductive folding displacement sensor (PSIFS) that used common paper material and copper tape was proposed for monitoring human respiration in [96].

### 4.2. Sensing Materials

Wearable sensors rely on various materials to enable their sensing capabilities and enhance their overall performance. These materials are carefully chosen based on their specific properties and the requirements of the sensor design. The conductivity of surfaces plays a critical role in the operation of various respiration sensors, such as resistive, capacitive, inductive, and antenna-based sensors, as these sensors depend on the surface’s conductivity to facilitate precise detection and measurements.

#### 4.2.1. Metallic Materials

Metallic materials are used in sensors due to their excellent electrical conductivity, which enables efficient signal transmission and sensing capabilities. For instance, a high-frequency litz wire was integrated into a textile shirt to make a coil for magnetic induction sensor in [94]. Furthermore, the utilization of an ultrathin metal film on a textured metallic disk can enable the detection of multipolar spoof localized surface plasmons for breathing monitoring [46]. Another approach involves a fully embroidered meander dipole antenna-based sensor integrated into a T-shirt, where the conductive part of the antenna sensor is composed of a silver-coated nylon thread [24]. Similarly, a wearable strain sensor for biomedical monitoring was developed in [28] employing a conductive layout knitted with highly conductive silver-coated yarns, resulting in a flexible and conductive fabric. Lastly, wearable sensors for non-contact monitoring of respiratory and heartbeat movements can employ conductive textiles as the active sensing material [48]. The sensor in this paper consisted of two layers of conductive textiles on a cloth substrate, with the top layer patterned as a disk-like resonator and the bottom layer acting as a ground plane.

In addition, conductive inks and paints have emerged as effective materials for the printing of sensors in the field of wearable technology. These offer numerous advantages, including flexibility, low-cost fabrication, and compatibility with various substrates. Silver conductive ink has been used to create sensing areas on nonwoven fabrics, enabling seamless integration into everyday clothing for textile-based respiration sensors [70,71]. Moreover, composite inks composed of elastomers, carbon particles, and PDMS microbeads have been used for printable textile-based strain and humidity sensors, offering conductivity as well as flexibility [80,83]. In [36], a temperature sensor for a smart bandage was fabricated by forming electrodes with conductive silver paint on a flexible PVC substrate. Thus, metallic materials play an indispensable role in the sensor systems by either contributing to sensing mechanisms or providing interconnects.

#### 4.2.2. Carbon-Based Materials

Various types of carbon materials, including carbon nanotubes, graphene, and MXene, have been used to fabricate wearable sensors for respiration sensors. Carbon nanotubes are cylindrical structures made of carbon atoms arranged in a hexagonal lattice with diameters on the scale of a nanometer, and are classified into two main types: single-walled carbon nanotubes (SWCNTs), and multi-walled carbon nanotubes (MWCNTs). To address the need for cost-effective and non-invasive respiratory monitoring, researchers have explored the use of multi-walled carbon nanotubes (MWCNTs) and polydimethylsiloxane (PDMS) composites [58,100,101]. These materials are utilized in the development of low-cost and reliable wearable sensors for human respiration monitoring. The integration of MWCNTs and PDMS in paper-based sensors offers an alternative solution to existing systems that require direct contact with the body and expensive monitoring equipment [62]. Furthermore, a biocompatible and lightweight respiration sensor consisting of highly oriented carbon nanotube (HO-CNT) films embedded between polyacrylonitrile (PAN) layers with excellent flexibility and robustness was presented in [121]. Moreover, a combination of lignin-based polyurethane foam and multi-walled carbon nanotubes (MWCNTs) has been used to provide conductivity and large surface area [91]. Additionally, MWCNTs have found application in self-powered wireless humidity sensors, showcasing an extensive range of humidity detection capabilities [122] and potential applications in respiration sensing.

Another popular carbon-based material is graphene, which is a single layer of carbon atoms arranged in a two-dimensional honeycomb lattice. It is employed in breathing analysis due to remarkable properties such as biocompatibility, low cost, and ease of functionalization [34]. In addition, MXene, which is a conductive and hydrophilic material belonging to a class of two-dimensional inorganic compounds, has been used for respiration sensing. MXene has been integrated into a melamine sponge (MS) structure to create a 3D bifunctional flexible sensor [82]. This MXene-based sensor exhibited excellent response to pressure and humidity, making it a versatile choice for respiration sensing applications. To achieve mechanical robustness and high operational stability under dry and wet conditions, researchers have employed composite inks composed of polyurethane elastomer, carbon black nanoparticles, poly(3-hexylthiophene-2,5-diyl) (P3HTs), and PDMS microbeads [80]. These flexible textile strain sensors offer promising capabilities for integration into wearable applications for breath monitoring.

The electrical and mechanical characteristics of commonly used sensing materials are summarized in Table 2.

#### 4.2.3. Conductive Polymers

Conductive polymers have emerged as promising materials for sensor applications due to their unique combination of electrical conductivity, mechanical flexibility, and tunable properties. For instance, a combination of polyethylene-co-vinyl acetate (PEVA) polymer and multi-walled carbon nanotubes (MWCNTs) has been utilized for antenna fabrication [25] for respiratory strain sensing. Multi-material fibers are another approach for developing respiration sensors. These fibers combine different materials to achieve enhanced performance and functionality in sensing applications. For respiration sensors, polyimide-coated hollow-core silica glass capillaries with a silver layer have been utilized as multi-material fibers [26,31], providing both the mechanical flexibility of polymers and the conductive properties of silver. In another study, poly(3,4-ethylenedioxythiophene) polystyrene sulfonate (PEDOT:PSS) polymer, which is a transparent conductive polymer with high ductility, was proposed for the design of a flexible strain sensor [36]. Such conductive polymers can be integrated into antenna designs and other sensing applications to enable accurate respiration monitoring.

#### 4.2.4. Piezoelectric Materials

Various piezoelectric materials have been employed in sensing due to their ability to generate electrical signals in response to mechanical pressure or strain. Materials such as polyvinylidene fluoride (PVDF) film and lead zirconate titanate (PZT) film have been used as sensing materials in respiration sensors. PVDF films can be utilized in nasal sensors for non-invasive respiratory monitoring [104], and have been employed as the piezoelectric material in self-powered active sensors for respiration monitoring [102]. A piezoelectric PZT thin film was utilized in a multifunctional wearable device that monitors motion and respiratory frequency in [97]. The development of flexible hybrid nanogenerators has included the incorporation of materials such as fluorinated ethylene propylene (FEP), nanocomposite of barium titanate (BaTiO3), and MWCNTs, enabling energy harvesting in the context of healthcare monitoring [100]. In [101], a battery-free electronic skin was proposed that combines PDMS and MWCNTs-doped PVDF for multi-modal sensing. PVDF has been extensively utilized in self-powering smart textile-based harvesters and sensors for the purpose of respiratory monitoring [103].

#### 4.2.5. Optical Fibers

Optical fibers are thin, flexible, and transparent strands made from high-quality glass or plastic materials that can transmit light signals over long distances with minimal loss. They have been extensively utilized in Fiber Bragg Grating (FBG) sensors used for strain sensing. These sensors are based on the principle of periodic variation in refractive index along an optical fiber, and offer unique advantages such as compatibility with magnetic resonance imaging (MRI) and immunity to electromagnetic interference [54]. Small and lightweight FBG sensors enable integration into wearable systems such as elastic belts, ensuring comfort and flexibility for users [56]. Usually, FBG sensors need to be encapsulated into flexible materials such as silicone rubber to enhance their practicality and usability in wearable devices intended for respiratory rate and heart rate monitoring applications [53,57].

### 4.3. Fabrication Techniques

Several existing fabrication techniques have been utilized to design functional and reliable sensors for a range of wearable applications. This subsection summarizes common fabrication techniques employed for wearable sensors, focusing on their application in breath monitoring.

#### 4.3.1. Printing

**Figure 9:**
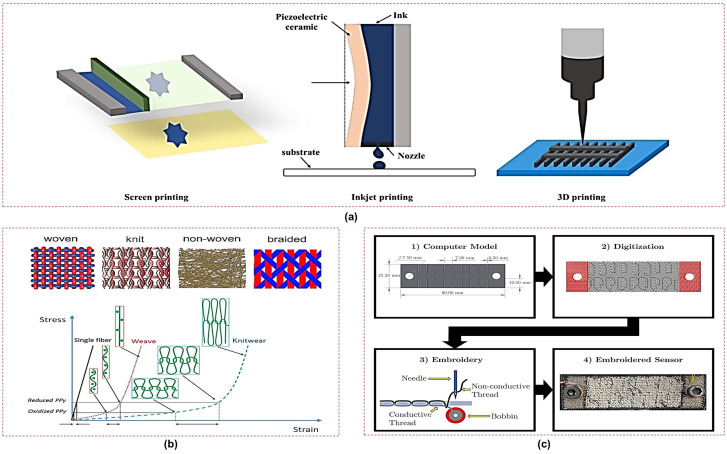
Fabrication techniques used for respiratory sensors: (**a**) printing [123]; (**b**) knitting [124]; (**c**) embroidery [125].

Various printing techniques, including screen printing, inkjet printing, and 3D printing, are employed in the fabrication of wearable sensors, as shown in Figure 9a. Screen printing is a frequently used technique in the fabrication of wearable sensors. It involves the deposition of conductive inks onto substrates through a mesh screen. This technique enables the creation of sensor electrodes, interconnections, and routing with high precision and repeatability. The use of conductive inks based on silver or carbon ensures good electrical conductivity while maintaining flexibility. Screen printing has been applied in the fabrication of capacitive sensors for respiration monitoring, where the ink is selectively deposited to create electrode patterns on the sensor substrate [62,63]. Moreover, screen printing is equally feasible for non-woven textiles and substrates, as demonstrated in [70,71]. Similarly, textile strain sensors can be fabricated using the screen printing process, in which the composite ink is printed onto a stretchable fabric to create the sensor [80]. This technique offers the advantage of scalability and cost-effectiveness, making it suitable for large-scale production of wearable sensors.

Other printing techniques, such as inkjet printing and 3D printing, are employed in wearable sensor fabrication. Inkjet printing allows for the precise deposition of functional materials such as conductive inks or graphene oxide solutions onto substrates to create sensors with high resolution and customization capabilities. The droplet-based nature of inkjet printing enables the creation of intricate sensor designs and the integration of multiple sensing elements on a single substrate. This technique has been utilized in articles where conductive inkjet printing is employed to fabricate wearable sensors [40,41]. On the other hand, 3D printing enables the fabrication of complex sensor structures and the integration of different materials. Using layer-by-layer deposition of materials, 3D printing allows for the creation of sensor components with varying mechanical properties and functionalities. This technique has been utilized for the fabrication of various parts of respiration wearable sensors [57,78]. Altogether, these printing techniques offer high resolution and design freedom, enabling the development of customized wearable sensors with unique geometries and functionalities.

#### 4.3.2. Knitting

Knitting and weaving techniques are employed in the fabrication of wearable sensors using conductive fibers or yarns. These techniques allow for the integration of conductive elements directly into the textile substrate, enabling seamless and flexible sensors. Conductive fibers or yarns such as metal-coated fibers or carbon nanotube yarns are incorporated during the knitting or weaving process to create conductive paths within the textile structure, as depicted in Figure 9b. For instance, a wearable antenna was fabricated using conductive yarns and knitting techniques in [28], with good conductivity ensured by a 99% pure silver-plated nylon yarn with tightly knitted loops. Similarly, a wearable system that integrated fabric sensors, electrodes, and connections all made using knitting techniques was proposed for remote health monitoring in [110]. In [67], the fabrication of a multifunctional e-textile incorporating polypyrrole on a knitted cotton/spandex fabric was proposed.

Additionally, CNC knitting machines are employed to seamlessly produce and integrate various components in a single knitting process, including sensors, antennas, conductive fabrics, nonconductive fabrics, and in one case a PCB pockets for a batteryless wearable compression sensor [16]. Moreover, circular knitting machines are used to design fabric electrodes for respiratory monitoring, enabling accurate measurements of resistance under different conditions [108]. In [76], a combination of knitting technology and coating printing industrial processes was utilized to fabricate knitted and printed sensors for a wearable monitoring system. The knitting and weaving approaches offer the advantage of simultaneous sensor fabrication and textile production, eliminating the need for additional assembly steps.

#### 4.3.3. Embroidery

Embroidery has been utilized in the fabrication of wearable sensors for various applications, including real-time breath monitoring. In this technique, conductive threads or yarns are embroidered onto the textile substrate to create sensor electrodes and connectors. The electrical conductivity of the threads can be optimized for desired sensor performance by carefully controlling the stitching pattern and density. For instance, an embroidered antenna-based sensor is proposed for real-time breathing monitoring using a commercial two-ply conductive yarn [30]. The layout of the embroidered meander dipole antenna sensor was converted into a digital stitch pattern and embroidered on a commercially available t-shirt using an embroidery machine, as illustrated in Figure 9c. This system successfully demonstrated real-time wireless monitoring of breathing patterns.

Similarly, a textile-based embroidered passive strain and displacement sensor was developed using standard embroidery processes and commercially available conductive yarns in [33]. Lastly, a wearable sensor for detecting respiration was created by embroidering conductive thread onto the fabric to form the electrodes and the connection terminals and circuit in [66]. The integration of conductive elements through embroidery allows for comfortable and unobtrusive sensor placement on clothing or other wearable garments. This technique offers several advantages, including the ability to create flexible and stretchable sensors that can conform to the shape of the human body.

## 5. Conclusions and Perspectives

This review has shown that low-frequency and high-frequency sensors can both be effectively utilized in respiration sensing, each offering unique advantages while facing specific challenges. Low-frequency sensors provide direct measurements of respiratory movements with simple designs and wide frequency response. However, challenges exist in terms of hysteresis, electrode-to-skin contact, motion artifacts, and environmental interference. On the other hand, high-frequency sensors offer wireless and contactless operation with good communication capabilities, while challenges arise in terms of power consumption, data transmission, and complexity of design. Furthermore, it is apparent that a highly diverse range of materials can be employed in the design of respiration sensors, ranging from polymers and textiles to metals and carbon-based substances. Obviously, these materials all possess their respective advantages and limitations in the context of specific applications. Therefore, key considerations should include materials with key properties such as flexibility and biocompatibility that can ensure long-term operation without causing discomfort to the user. Moreover, commonly used fabrication techniques such as printing, knitting, and embroidery have been discussed and assessed based on factors such as the design resolution of the sensor, manufacturing cost, and suitability for large-scale production.

In the context of future research, promising potential exists for self-powered wearable respiration sensors specifically designed for low-frequency applications. These sensors utilize piezoelectric and triboelectric components to create self-sustaining systems. However, challenges persist in miniaturization and efficiently when converting mechanical breathing energy into electrical power while ensuring user comfort. High-frequency sensors have emerged as a promising trend in the field of respiration sensors due to their ability to simultaneously sense and communicate breathing information in a compact form factor. In this context, antenna sensors and RFID sensors have shown promise as viable candidates for this purpose. Additional research could focus on enhancing the design of wearable antenna sensors to minimize radiation exposure to users. Furthermore, exploring chipless RFID sensors as an alternative to chip-based RFID could facilitate larger-scale production and ensure long-term operation. The integration of metamaterials into existing sensors holds great promise, offering advantages such as tunability and enhanced sensing capabilities. Additionally, as flexible metamaterials are very versatile on their own, they could be used for sensing, shielding, and energy harvesting in smart textiles. Opportunities for further research can be found in the optimization of metamaterial design and fabrication techniques that can enable their seamless integration into wearable devices for diverse sensing applications, including respiration monitoring.

## Figures and Tables

**Table 1:** Properties of substrates used for respiration sensors.

Substrate	Mechanical Properties	Biocompatibility
Polydimethylsiloxane (PDMS)	Soft, conformable	Excellent
Polyurethane (PU)	Soft, flexible	Good
Polyimide (PI)	High tensile strength, flexible	Good
Hydrogels/Ion gels	Soft, stretchable	Good
Rubbers (e.g., Ecoflex)	Soft, similar modulus to human skin	Good
Polyvinylidene Fluoride (PVDF)	High mechanical strength, flexibility, low density	Good
Fabric Substrate (e.g., cotton, wool, lycra)	Flexible, stretchable, comfortable	Excellent
Stretchable Fabric	Stretchable, resilient	Excellent
Knitted Fabric (e.g., cotton/spandex)	Elastic, flexible	Excellent
Nonwoven Fabric	Lightweight, flexible	Excellent
Lignin-based Polyurethane Foam	Elastic, lightweight	Good
Cellulose paper	Brittle, low tensile strength	Good
Filter paper	Porous, flexible	Good

**Table 2:** Properties of sensing materials used in respiration sensors.

Metallic Material	Electrical Conductivity	Mechanical Properties
Silver-plated nylon yarn	Excellent	Flexible, soft, conformable
Conductive Textile and Fabrics	Excellent	Flexible, stretchable, comfortable
Conductive inks	Good	Flexible, adaptable
Polyethylene-co-vinyl acetate (PEVA)	Conductive with MWCNTs	Highly flexible
Polyimide-coated hollow-core silica glass capillaries with silver layer	Conductive with silver layer	Highly flexible
Multi-walled Carbon Nanotubes (MWCNTs)	Excellent	Flexible, high tensile strength
Graphene	High	Flexible, strong
MXene	Good	Flexible, mechanically robust
Carbon black nanoparticles	Good	Flexible, good resilience
Polyvinylidene Fluoride (PVDF)	Piezoelectric properties	Flexible, strong

## Data Availability

No new data were created.
